# A targetable pathway to eliminate TRA-1-60^+^/TRA-1-81^+^ chemoresistant cancer cells

**DOI:** 10.1093/jmcb/mjad039

**Published:** 2023-06-16

**Authors:** Lei Tan, Xiaohua Duan, Pratyusha Mutyala, Ting Zhou, Sadaf Amin, Tuo Zhang, Brian Herbst, Gokce Askan, Tomer Itkin, Zhaoying Xiang, Fabrizio Michelassi, Michael D Lieberman, Christine A Iacobuzio-Donahue, Steven D Leach, Todd Evans, Shuibing Chen

**Affiliations:** Department of Surgery, Weill Cornell Medicine, New York, NY 10065, USA; Center for Energy Metabolism and Reproduction, Institute of Biomedicine and Biotechnology, Shenzhen Institute of Advanced Technology, Chinese Academy of Sciences, Shenzhen 518055, China; Department of Surgery, Weill Cornell Medicine, New York, NY 10065, USA; Center for Genomic Health, Weill Cornell Medicine, New York, NY 10065, USA; Department of Surgery, Weill Cornell Medicine, New York, NY 10065, USA; The SKI Stem Cell Research Facility, The Center for Stem Cell Biology and Developmental Biology Program, Memorial Sloan Kettering Cancer Center, New York, NY 10065, USA; Department of Surgery, Weill Cornell Medicine, New York, NY 10065, USA; Genomic Resource Core Facility, Weill Cornell Medical College, New York, NY 10065, USA; Rubenstein Center for Pancreatic Cancer Research, Memorial Sloan Kettering Cancer Center, New York, NY 10065, USA; Rubenstein Center for Pancreatic Cancer Research, Memorial Sloan Kettering Cancer Center, New York, NY 10065, USA; Division of Regenerative Medicine, Hartman Institute for Therapeutic Organ Regeneration, Ansary Stem Cell Institute, Department of Medicine, Weill Cornell Medicine, New York, NY 10065, USA; Genomic Resource Core Facility, Weill Cornell Medical College, New York, NY 10065, USA; Department of Surgery, Weill Cornell Medicine, New York, NY 10065, USA; Department of Surgery, Weill Cornell Medicine, New York, NY 10065, USA; Rubenstein Center for Pancreatic Cancer Research, Memorial Sloan Kettering Cancer Center, New York, NY 10065, USA; Dartmouth Cancer Center, Darmouth College, Hanover, NH 03755, USA; Department of Surgery, Weill Cornell Medicine, New York, NY 10065, USA; Center for Genomic Health, Weill Cornell Medicine, New York, NY 10065, USA; Department of Surgery, Weill Cornell Medicine, New York, NY 10065, USA; Center for Genomic Health, Weill Cornell Medicine, New York, NY 10065, USA

**Keywords:** pancreatic cancer, TRA-1-60/TRA-1-81, chemoresistance, UGT1A10, Cymarin

## Abstract

Chemoresistance is a primary cause of treatment failure in pancreatic cancer. Identifying cell surface markers specifically expressed in chemoresistant cancer cells (CCCs) could facilitate targeted therapies to overcome chemoresistance. We performed an antibody-based screen and found that TRA-1-60 and TRA-1-81, two ‘stemness’ cell surface markers, are highly enriched in CCCs. Furthermore, TRA-1-60^+^/TRA-1-81^+^ cells are chemoresistant compared to TRA-1-60^–^/TRA-1-81^–^ cells. Transcriptome profiling identified *UGT1A10*, shown to be both necessary and sufficient to maintain TRA-1-60/TRA-1-81 expression and chemoresistance. From a high-content chemical screen, we identified Cymarin, which downregulates *UGT1A10*, eliminates TRA-1-60/TRA-1-81 expression, and increases chemosensitivity both *in vitro* and *in vivo*. Finally, TRA-1-60/TRA-1-81 expression is highly specific in primary cancer tissue and positively correlated with chemoresistance and short survival, which highlights their potentiality for targeted therapy. Therefore, we discovered a novel CCC surface marker regulated by a pathway that promotes chemoresistance, as well as a leading drug candidate to target this pathway.

## Introduction

Pancreatic cancer is one of the most challenging solid tumors, with a 5-year survival rate of 7.2%. The major treatment option for unresectable/metastatic pancreatic cancer is chemotherapy, which can be combined with surgery as neoadjuvant or adjuvant therapy. Gemcitabine (GEM), either by itself or in combination with other drugs, is considered a first-line agent for locally advanced and metastatic pancreatic cancer to improve quality of life and prolong survival. However, outcome remains poor due to a high rate of chemoresistance (Ying et al., 2012).

Antigen-specific targeted therapy is potentially effective to enhance treatment by specifically eliminating chemoresistant cancer cells (CCCs), as developed to treat leukemia. For example, CD126 blocking antibody enhanced chemotherapy on chemoresistant chronic lymphocytic leukemia cells, which display active signaling through the IL6/CD126/STAT3 axis (Liu et al., 2015). In clinical trials, Gemtuzumab ozogamicin, the conjugation of anti-CD33 antibody and calicheamicin, improved survival of acute myeloid leukemia patients (Burnett et al., 2011, 2012; Castaigne et al., 2012; Hills et al., 2014). However, few antigen-specific targeted therapies have been tested to conquer chemoresistance of solid tumors, including pancreatic cancer. One of the obstacles is to identify specific cell surface marker(s) that label CCCs.

Several cell surface markers have been reported to enrich chemoresistant pancreatic cancer cells (Hermann et al., 2007; Shah et al., 2007; Hong et al., 2009; Avan et al., 2013; Ioannou et al., 2016). For example, CD133^+^ cancer stem cells show high resistance to standard chemotherapy (Hermann et al., 2007); CD44 sustains GEM resistance in pancreatic cancer cells (Hong et al., 2009); c-MET is also enhanced in GEM resistant (GR) pancreatic cancer cells and necessary for chemoresistance (Shah et al., 2007; Li et al., 2011; Avan et al., 2013; Ioannou et al., 2016). Both chemical (Quan et al., 2013; Schott et al., 2013; Kim et al., 2014) and antibody-based (Salnikov et al., 2013; Li et al., 2014; Arabi et al., 2015; Schmohl et al., 2015; Amoury et al., 2016) strategies have been evaluated to target cell surface markers, and some phase I clinical trials have proceeded for CD44 (Lin et al., 2015) and c-MET (Oguri et al., 2004; Pant et al., 2014) in pancreatic cancer. In these trials, a major potential problem is that the markers being targeted are widely expressed in healthy tissues. For example, CD44 is highly expressed in lymphocytes (de la Hera et al., 1989), CD133 is a hematopoietic stem/progenitor cell marker (Kobari et al., 2001), and c-Met is extensively expressed in the liver, gastro-intestinal tract, and kidney (Prat et al., 1991). Expression of these antigens in healthy tissues raises the concern of ‘off-target’ effects in ‘targeted’ therapy.

Here, we report an antibody-based cell surface marker screen that identified TRA-1-60 and TRA-1-81 antibodies recognizing an antigen enriched in chemoresistant pancreatic ductal adenocarcinoma (PDAC) cell lines and primary patient samples. TRA-1-60^+^/TRA-1-81^+^ cells are chemoresistant compared to the negative cohort. Global transcriptional profiling identified UGT1A10 as a necessary and sufficient regulator of TRA-1-60/TRA-1-81 expression and chemoresistance. Finally, a high-content chemical screen discovered Cymarin (CYM), an anti-arrhythmia agent, as a lead drug that decreases *UGT1A10* expression, eliminates TRA-1-60^+^/TRA-1-81^+^ cells, and increases chemosensitivity both *in vitro* and *in vivo*.

## Results

### The common epitope of TRA-1-60 and TRA-1-81 antibodies is highly enriched in CCCs

**Figure 1:**
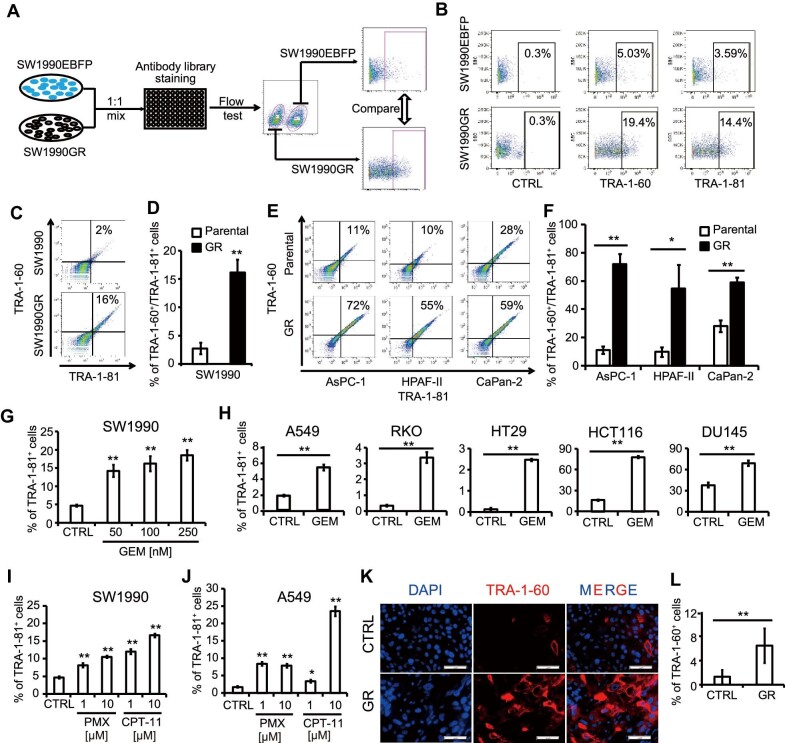
TRA-1-60 and TRA-1-81 are enriched in CCCs. (**A**) Scheme of the antibody-based cell surface marker screen. See also Supplementary Figure S1A. (**B**) Flow cytometry plots of TRA-1-60 and TRA-1-81 expression in the primary screen. See also Supplementary Figure S1B. (**C** and **D**) Flow cytometry plots (**C**) and quantification (**D**) of TRA-1-60 and TRA-1-81 co-staining in SW1990 and SW1990GR cells. (**E** and **F**) Flow cytometry plots (**E**) and quantification (**F**) of TRA-1-60 and TRA-1-81 co-staining in multiple pancreatic cancer cell lines and their GR derivatives. (**G**) The percentage of cells in the TRA-1-81^+^ population in SW1990 cells after acute GEM treatment. See also Supplementary Figure S1C. (**H**) The percentage of cells in the TRA-1-81^+^ population in a panel of cancer cell lines after acute GEM treatment. See also Supplementary Figure S1D. (**I**) The percentage of cells in the TRA-1-81^+^ population in SW1990 cells after acute treatment of PMX or CPT-11. See also Supplementary Figure S1E (upper). (**J**) The percentage of cells in the TRA-1-81^+^ population in A549 cells after acute treatment of PMX or CPT-11. See also Supplementary Figure S1E (lower). (**K** and **L**) Immunohistochemistry staining (**K**) and quantification (**L**) of TRA-1-60 in control SW1990 and SW1990GR xenograft tumors. Values in graphs are represented as mean ± standard error of the mean (SEM). N = 3 independent biological replicates. Scale bar, 50 μm. *P*-values by unpaired two-tailed student's *t*-test were *P < 0.05 and **P < 0.01.

To develop a screening platform, we first established a GR cell line SW1990GR (Supplementary Figure S1A). The IC_50_ for GEM of SW1990GR (59.4 ± 5.3 nM) is ∼6 times higher than parental SW1990 cells (9.6 ± 3.2 nM). Screening was then carried out to identify candidate markers correlated with chemoresistance of cancer cells (Figure 1A). The parental line expressing an enhanced blue fluorescent protein (EBFP) reporter (SW1990EBFP) and SW1990GR were mixed and stained with a human cell surface marker antibody library. The positive percentage of each marker in the two cell lines was quantified and further analyzed in two-dimensional space. As shown in Supplementary Figure S1B, the x-axis represents the absolute difference value, obtained by subtracting the positive percentage in SW1990EBFP cells from that in SW1990GR cells; the y-axis indicates the fold change, obtained by dividing the positive percentage in SW1990GR cells by that in SW1990EBFP cells. As highlighted, eight antigens were preferentially expressed in SW1990GR cells (Supplementary Figure S1B). Among them was CD24, a marker of pancreatic cancer stem cells (Li et al., 2007) and regulator of drug resistance in breast cancer cell lines (Lim et al., 2014), which validated our screening platform. After several rounds of validation, TRA-1-60- and TRA-1-81-expressing cells were confirmed enriched in the SW1990GR cell line (Figure 1B).

As shown in a previous report based on glycan array screening, TRA-1-60 and TRA-1-81 antibodies bind to the same polysaccharide epitope with high affinity (Natunen et al., 2011). To confirm this correlation, SW1990 and SW1990GR cells were co-stained with TRA-1-60 and TRA-1-81 antibodies. The two antigens showed strong co-staining in flow cytometry and identified the same cell population significantly enriched in SW1990GR cells (16.5% ± 2.7%) compared to SW1990 cells (2.8% ± 1.3%) (Figure 1C and D). Therefore, we used the two markers interchangeably for subsequent experiments.

To determine whether this epitope is enriched in other GR PDAC cell lines, AsPC-1GR, HPAF-IIGR, CaPan-2GR, and their corresponding parental cell lines were analyzed via flow cytometry. The TRA-1-60^+^/TRA-1-81^+^ cell population was significantly larger in all three GR cell lines compared with corresponding parental lines (Figure 1E and F). To examine whether TRA-1-60 and TRA-1-81 are enriched in PDAC cells after acute treatment, SW1990 cells were treated with 50, 100, or 250 nM GEM for 5 days. Flow cytometry analysis indicated a significant increase of TRA-1-81^+^ cells in GEM-treated SW1990 cells (Figure 1G; Supplementary Figure S1C). We also examined other cancer cell lines A549, RKO, HT29, HCT116, and DU145. Consistent with the results in SW1990 cells, a significant increase of TRA-1-81^+^ cell population was detected after acute GEM treatment (Figure 1H; Supplementary Figure S1D). We tested whether the TRA-1-60 and TRA-1-81 epitope is enriched in PDAC cells resistant to other chemotherapeutic agents, after treating SW1990 cells with PMX (Miller et al., 2000; Oettle et al., 2005) or CPT-11 (Sakata et al., 1994; Wagener et al., 1995) for 5 days. Similar to the effects of GEM, flow cytometry showed that the TRA-1-81^+^ cell population was highly enriched in PMX- or CPT-11-treated SW1990 cells (Figure 1I; Supplementary Figure S1E). Additionally, the A549 cell line also exhibited a significant enhancement of TRA-1-81^+^ portion after acute CPT-11 or PMX treatment (Figure 1J; Supplementary Figure S1E).

We then examined whether the epitope of TRA-1-60 and TRA-1-81 antibodies also enriches CCCs *in vivo*, with xenograft tumors in nude mice derived from SW1990 cells implanted subcutaneously. Mice carrying tumors were randomly separated into two groups: one was treated with 50 mg/kg GEM via intraperitoneal injection twice a week for 4 weeks; the other was treated with the same volume of phosphate-buffered saline (PBS) as control. After three passages *in vivo* (∼6 months), the control and GR xenograft tumors were analyzed with immunofluorescence (Figure 1K). The percentage of TRA-1-60^+^ cells was significantly increased in the GR xenograft tumors (Figure 1L). Together, these experiments demonstrate that TRA-1-60 and TRA-1-81 are surface markers that significantly enrich CCCs both *in vitro* and *in vivo*.

### TRA-1-60^+^/TRA-1-81^+^ cells are resistant to GEM treatment

**Figure 2:**
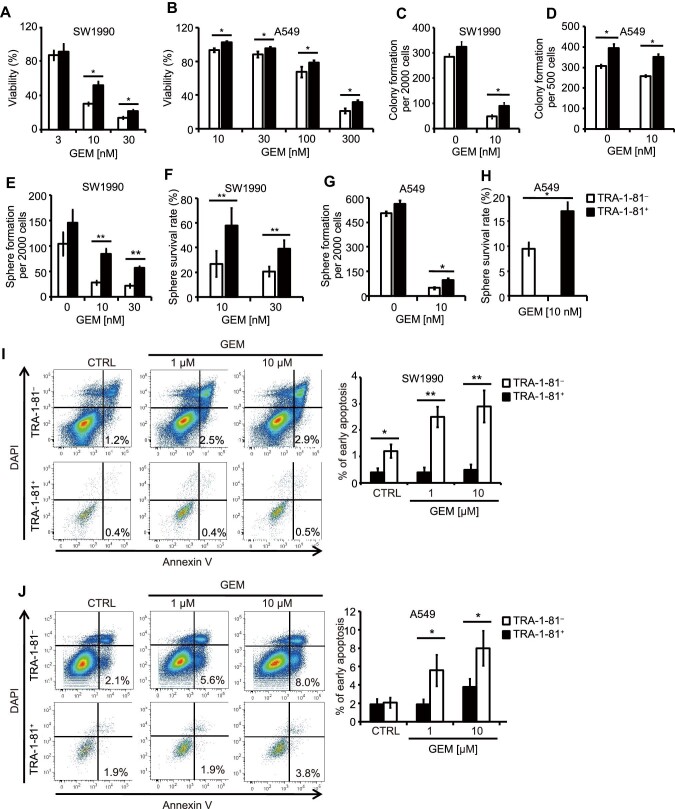
TRA-1-60^+^/TRA-1-81^+^ cells show higher resistance to GEM treatment. (**A**) Viability of TRA-1-60^+^/TRA-1-81^+^ cells (black bars) and TRA-1-60^–^/TRA-1-81^–^ cells (white bars) isolated from SW1990 cells cultured in the presence of GEM. (**B**) Viability of TRA-1-81^+^ and TRA-1-81^–^ cells isolated from A549 cells cultured in the presence of GEM. (**C** and **D**) Colony formation of TRA-1-81^+^ and TRA-1-81^–^ populations isolated from SW1990 cells (**C**) or A549 cells (**D**) cultured in the absence or presence of GEM. See also Supplementary Figure S2A and B. (**E**–**H**) The number of spheres derived from TRA-1-81^+^ and TRA-1-81^–^ cells isolated from SW1990 cells (**E**) and A549 cells (**G**) cultured in the absence or presence of GEM. See also Supplementary Figure S2C and D. Sphere survival rate of SW1990 cells (**F**) and A549 cells (**H**) was calculated by dividing the sphere number in GEM-treated condition by that in control condition. (**I** and **J**) Flow cytometry analysis and quantification of Annexin V^+^ cells in SW1990 cells (**I**) and A549 cells (**J**) treated with or without GEM. Values in graphs are represented as mean ± SEM. N = 3 independent biological replicates. *P*-values by unpaired two-tailed student's *t*-test were *P < 0.05 and **P < 0.01.

TRA-1-60^+^/TRA-1-81^+^ cells were examined for survival, colony formation, and sphere formation following GEM treatment. First, TRA-1-81^+^ and TRA-1-81^–^ cells isolated from the SW1990 cell line were treated with GEM at different concentrations (3, 10, or 30 nM) for 5 days. Relative cell viability was calculated by normalizing cell numbers in GEM-treated conditions to that in control conditions. TRA-1-81^+^ cells showed higher viability than TRA-1-81^–^ cells treated with 10 or 30 nM GEM (Figure 2A). In addition, the TRA-1-81 expression level of TRA-1-81^+^ SW1990GR cells is significantly higher than that of TRA-1-81^+^ SW1990 cells (Supplementary Figure S2A). Similarly, TRA-1-81^+^ A549 cells showed higher survival than TRA-1-81^–^ cells following GEM treatment (Figure 2B). Second, colony formation was used to monitor single cell survival under GEM treatment. In both SW1990 and A549 cell lines, the TRA-1-81^+^ population yielded more colonies (Figure 2C and D; Supplementary Figure S2B and C). Third, sphere formation was evaluated in the presence of GEM. For both cell lines, more spheres were generated by TRA-1-81^+^ cells than TRA-1-81^–^ cells in both control and GEM-treated conditions (Figure 2E and G; Supplementary Figure S2D and E). Sphere survival rate was calculated by dividing sphere number in the presence of GEM by sphere number in control conditions. TRA-1-81^+^ cells showed significantly higher sphere survival rate than TRA-1-81^–^ cells (Figure 2F and H).

Cell apoptosis induced by GEM was monitored with Annexin V staining. For both SW1990 cells (Figure 2I) and A549 cells (Figure 2J), the percentages of Annexin V^+^/DAPI^–^ (early apoptotic) cells were significantly higher in TRA-1-81^–^ populations in a GEM dose-dependent manner. Meanwhile, early apoptotic cells in the TRA-1-81^+^ population were modestly induced, consistent with resistance to chemotherapy drug treatment. We further compared the early apoptotic rate among TRA-1-81^–^, TRA-1-81^medium^, and TRA-1-81^high^ populations in GR cells. The apoptotic rate of TRA-1-81^high^ cells was significantly lower than that of TRA-1-81^medium^ cells (Supplementary Figure S2F).

### Gene expression profiling identifies UGT1A10 as a functional mediator of TRA-1-60/TRA-1-81 expression and chemoresistance

**Figure 3:**
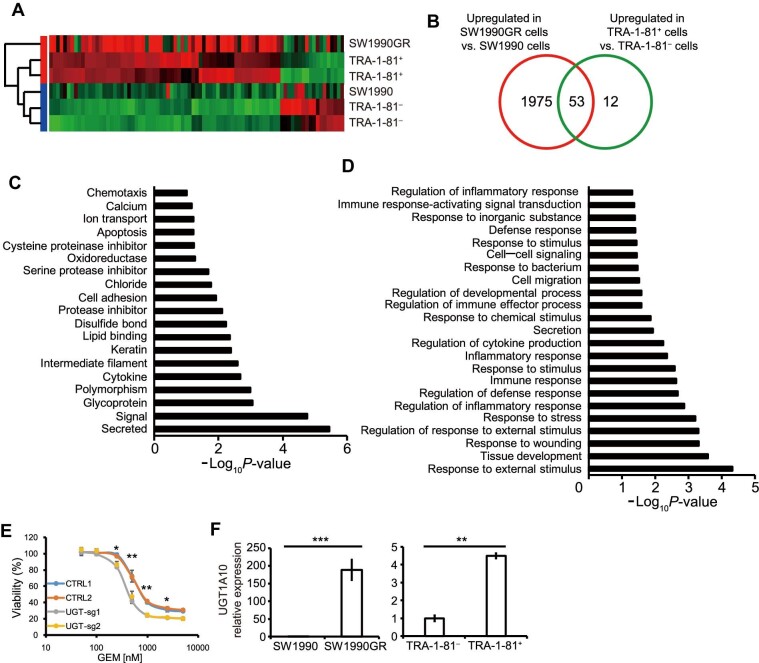
Gene expression profiling identifies UGT1A10 as a functional mediator of TRA-1-60/TRA-1-81 expression and chemoresistance. (**A**) Heatmap from gene expression profiling of SW1990 cells vs. SW1990GR cells and TRA-1-81^+^ cells vs. TRA-1-81^–^ cells in SW1990 cells. (**B**) Venn diagram of genes upregulated in SW1990GR cell line (compared with SW1990 cell line) and TRA-1-81^+^ cells (compared with TRA-1-81^–^ cells). (**C** and **D**) Ontology analysis of genes upregulated in both SW1990GR cells and TRA-1-81^+^ cells for protein properties (**C**) and functions (**D**). (**E**) Viability of SW1990GR cells expressing sgRNAs targeting *UGT1A10* in the presence of GEM treatment. See also Supplementary Figure S3. (**F**) qRT-PCR analysis of *UGT1A10* differential expression in SW1990 cells vs. SW1990GR cells (left) and in TRA-1-81^+^ vs. TRA-1-81^–^ SW1990 cells (right). N = 3 independent biological replicates for **E** and **F**. *P*-values by unpaired two-tailed student's *t*-test were *P < 0.05, **P < 0.01, and ***P < 0.005.

To interrogate the molecular basis for chemoresistance in TRA-1-60^+^/TRA-1-81^+^ cells, RNA sequencing was performed to compare the global transcriptomes between TRA-1-81^+^ and TRA-1-81^–^ cells sorted from the SW1990 cell line, and independently, between parental SW1990 and SW1990GR cells. This identified 65 genes that are upregulated at least 2-fold in TRA-1-81^+^ cells compared to TRA-1-81^–^ cells and 2028 genes that are elevated at least 3-fold in SW1990GR cells compared to parental SW1990 cells (Figure 3A). The gene subset commonly upregulated in both TRA-1-81^+^ and SW1990GR cells is comprised of 53 genes (Figure 3B). Gene ontology analysis was performed (Huangda et al., 2009) to classify this subset into distinct groups according to protein category (Figure 3C) and protein function (Figure 3D). We noted that glycoprotein is among the top group of proteins represented among these 53 shared genes (Figure 3C). Most genes within this shared set are involved in response to stimulus, stress, and substrate (Figure 3D), suggesting that they may impact robustness of survival in TRA-1-81^+^ cells when challenged with chemo-drugs.

From the shared gene set, seven candidates that showed relatively higher basal expression and higher fold change were selected for functional validation (Supplementary Table S1). Two sgRNAs were designed for each gene to target the first coding exon, and two non-targeting sgRNAs were designed as controls (see Supplementary material). After transducing sgRNA and Cas9 enzyme by lentivirus (Sanjana et al., 2014), SW1990GR cells were examined for chemoresistance. The cells with *UGT1A10* targeted (UGT-sg) showed significantly lower viability following GEM treatment (Figure 3E), while targeting the other six candidates showed no effect (Supplementary Figure S3A). Efficient reduction of UGT1A10 expression in UGT-sg cells was confirmed by western blotting experiments (Supplementary Figure S3B and C). Data from quantitative real-time polymerase chain reaction (qRT-PCR) experiments confirmed that *UGT1A10* mRNA levels are strikingly higher in SW1990GR and TRA-1-81^+^ cells (Figure 3F).

### UGT1A10 is both required and sufficient to maintain high levels of TRA-1-60^+^/TRA-1-81^+^ cells and enhance chemoresistance

**Figure 4:**
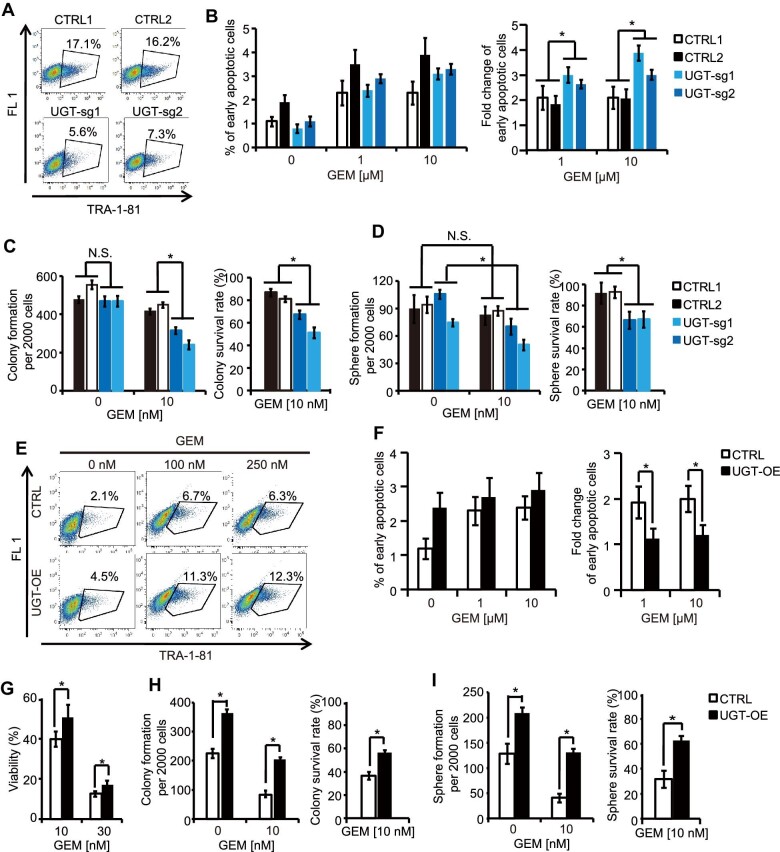
UGT1A10 enhances both TRA-1-60/TRA-1-81 expression and chemoresistance. (**A**) Flow cytometry plots of TRA-1-81 expression in UGT-sg SW1990GR cells. (**B**) Quantification of flow cytometry plots of Annexin V staining in UGT-sg cells. See also Supplementary Figure S4A. (**C** and **D**) Colony formation (**C**) and sphere formation (**D**) of UGT-sg SW1990GR cells. (**E**) Flow cytometry plots of TRA-1-81 expression in UGT-OE SW1990 cells. (**F–I**) Quantification of flow cytometry plots of Annexin V staining (**F**), viability (**G**), colony formation (**H**), and sphere formation (**I**) of UGTOE-SW1990 cells. The sensitivity to GEM treatment was determined by dividing the percentage of Annexin V^+^/DAPI^–^ early apoptotic cells in GEM-treated conditions by that in control condition. See also Supplementary Figure S4B. Values in graphs are represented as mean ± SEM. N = 3 independent biological replicates. *P*-values by unpaired two-tailed student's *t*-test were *P < 0.05 and **P < 0.01.

Flow cytometry analysis showed that the percentage of TRA-1-81^+^ cells was significantly decreased in UGT-sg cells, suggesting that UGT1A10 is required to maintain TRA-1-81 expression (Figure 4A). GEM sensitivity was monitored by comparing the percentage of Annexin V^+^/DAPI^–^ early apoptotic cells induced by GEM treatment with that induced by control treatment. UGT-sg cells showed a higher sensitivity to GEM than control SW1990GR cells (Figure 4B; Supplementary Figure S4A). Colony-forming capacity was also compared. In GEM-free conditions, there was no significant difference between UGT-sg cells and control SW1990GR cells. In GEM-treated conditions, numbers of colonies derived from UGT-sg cells were significantly lower that from control cells (Figure 4C, left panel). The colony survival rate was determined by normalizing colony number in the presence of GEM with the number in the absence of GEM, and UGT-sg cells showed a significantly decreased colony survival rate (Figure 4C, right panel), suggesting that knockout of *UGT1A10* impaired the robustness of colony-forming capacity. Finally, UGT-sg and control SW1990GR cells were cultured in suspension to examine their relative sphere-forming ability. The number of spheres produced by UGT-sg cells decreased dramatically in the presence of GEM. In contrast, the number of spheres produced by control cells was not significantly changed between GEM-treated and control conditions (Figure 4D). The sphere survival rate of UGT-sg cells was significantly lower than that of control cells. Therefore, UGT1A10 is necessary for both maintenance of TRA-1-81^+^ cells and chemoresistance.

To determine whether UTG1A10 is sufficient to induce TRA-1-81 expression and chemoresistance, *UGT1A10* was overexpressed ectopically in SW1990 cells (UGT-OE). Cells expressing firefly luciferase were used as control. A significant increase of TRA-1-81^+^ cells was detected in UGT-OE compared with control cells, either in the absence or presence of GEM (Figure 4E). While GEM treatment significantly increased the percentage of Annexin V^+^/DAPI^–^ early apoptotic cells (Figure 4F; Supplementary Figure S4B) in control cells, UGT-OE cells did not respond to GEM (Figure 4F, right). Indeed, the viability of UGT-OE cells is higher than that of control cells following GEM treatment (Figure 4G). Furthermore, UGT-OE cells showed less reduction of colony-forming (Figure 4H) and sphere-forming (Figure 4I) capacities after challenge by GEM. In summary, UGT1A10 is both required and sufficient to enhance TRA-1-60 and TRA-1-81 expression and chemoresistance.

### A high-content chemical screen identifies CYM as a compound that specifically eliminates TRA-1-60^+^/TRA-1-81^+^ cells and increases chemosensitivity *in vitro*

**Figure 5:**
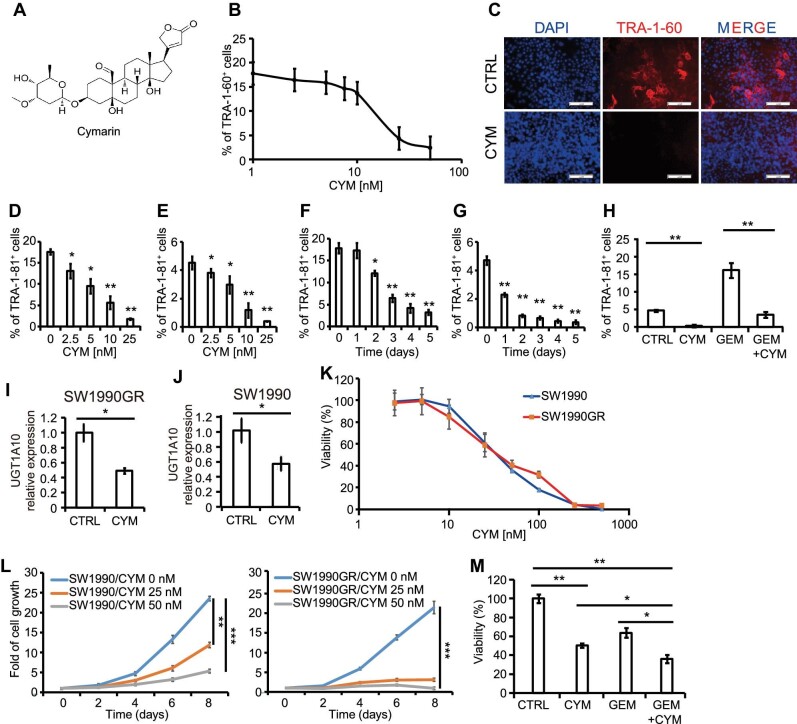
A high-content chemical screen identifies CYM as a compound that decreases the percentage of TRA-1-60^+^ cells and increases chemosensitivity. (**A**) Chemical structure of CYM. See also Supplementary Figure S5A and B. (**B**) TRA-1-60 inhibition curve of SW1990GR cells with CYM treatment, generated via immunofluorescence. See also Supplementary Figure S5C. (**C**) Immunofluorescence of TRA-1-60 staining in control or CYM-treated SW1990GR cells. (**D** and **E**) Dose curve of CYM on the percentage of TRA-1-81^+^ SW1990GR cells (**D**) and parental SW1990 cells (**E**), generated via flow cytometry. See also Supplementary Figure S5D. (**F** and **G**) Time course of CYM on the percentage of TRA-1-81^+^ SW1990GR cells (**F**) and parental SW1990 cells (**G**), generated via flow cytometry. See also Supplementary Figure S5E. (**H**) Quantification of TRA-1-81^+^ SW1990 cells treated with control, 100 nM GEM, 25 nM CYM, or 100 nM GEM + 25 nM CYM, generated via flow cytometry. See also Supplementary Figure S5G. (**I** and **J**) qRT-PCR analysis of *UGT1A10* expression in SW1990GR cells (**I**) and SW1990 cells (**J**) with CYM treatment. (**K**) Viability curves of SW1990 and SW1990GR cells with CYM treatment. (**L**) Growth curves of SW1990 (left) and SW1990GR (right) cells with CYM treatment. (**M**) Viability of SW1990 cells treated with control, 30 nM GEM, 25 nM CYM, or 30 nM GEM + 25 nM CYM. See also Supplementary Figure S5I. Values in graphs are represented as mean ± SEM. N = 3 independent biological replicates. Scale bar, 100 μm. *P*-values by unpaired two-tailed student's *t*-test were *P < 0.05 and **P < 0.01.

A high-content chemical screen was performed to identify drug candidates specifically eliminating TRA-1-60^+^/TRA-1-81^+^ cells. Since this antigen appears to mark CCCs, such a drug could possibly reduce chemoresistance. After treating SW1990GR cells with the chemical library, the percentage of TRA-1-60^+^ cells was assessed with immunocytochemistry. A total of 20 compounds that decreased the percentage of TRA-1-60^+^ cells by >12% were picked as primary hits (Supplementary Figure S5A). We were able to validate two of the hits, CYM (Figure 5A) and the structurally related Ouabain (Supplementary Figure S5B). We focused on CYM, since it showed higher potency than Ouabain (Supplementary Figure S5C).

CYM decreased the percentage of TRA-1-60^+^ cells in a dose-dependent manner based on immunofluorescent staining, with IC_50_ at 15.2 ± 4.3 nM (Figure 5B). After 5 days of treatment with 25 nM CYM, the percentage of TRA-1-60^+^ cells decreased from ∼18% to < 5% (Figure 5B and C). Flow cytometry analysis confirmed that CYM decreased the percentage of TRA-1-81^+^ SW1990GR cells in a dose-dependent manner, with IC_50_ at 5.1 ± 1.5 nM (Figure 5D; Supplementary Figure S5D). For the parental SW1990 cells, CYM also repressed TRA-1-81^+^ cells in a dose-dependent manner, with IC_50_ at 6.3 ± 2.2 nM (Figure 5E; Supplementary Figure S5D). To determine the minimal time period required to eliminate TRA-1-81^+^ cells, a time course experiment was performed and showed the percentage of TRA-1-81^+^ cells decreasing over time in both SW1990GR cells (Figure 5F; Supplementary Figure S5D) and SW1990 cells (Figure 5G; Supplementary Figure S5E). CYM also decreased the percentage of TRA-1-81^+^ cells in other cancer cell lines, e.g. prostate cancer cell line DU145 (Supplementary Figure S5F, left) and colorectal cancer cell line HCT-116 (Supplementary Figure S5F, right). To determine whether CYM attenuates the enrichment of TRA-1-81^+^ cells caused by acute GEM treatment, the percentage of TRA-1-81^+^ cells was evaluated by flow cytometry in SW1990 cells after 5 days of treatment with 100 nM GEM, 25 nM CYM, or 100 nM GEM plus 25 nM CYM. CYM treatment decreased the percentage of TRA-1-81^+^ cells effectively regardless of GEM (Figure 4H; Supplementary Figure S5G).

We considered that CYM may eliminate TRA-1-81^+^ cells either by killing TRA-1-81^+^ cells or through suppression of TRA-1-60/TRA-1-81 epitope expression. After 1-day treatment of 100 nM or 200 nM CYM, apoptosis was assessed in SW1990GR cells. The percentages of Annexin V^+^/DAPI^–^ (early apoptotic) cells and Annexin V^+^/DAPI^+^ (late apoptotic) cells were increased in both TRA-1-81^+^ and TRA-1-81^–^ subpopulations in a dose-dependent manner (Supplementary Figure S5H). However, the increasing trends of apoptosis showed little difference between TRA-1-81^+^ and TRA-1-81^–^ populations. Rather, qRT-PCR assays showed that *UGT1A10* transcript levels were decreased with CYM treatment in both SW1990GR (Figure 5I) and SW1990 (Figure 5J) cells. Therefore, most likely the reduction of TRA-1-60^+^/TRA-1-81^+^ cells caused by CYM is not due to targeted cell death, but due to the modulation of functional molecular targets, e.g. levels of UGT1A10.

To evaluate the effect of CYM on cell viability, both SW1990 and SW1990GR cells were cultured with CYM (concentration ranging 0–500 nM) for 3 days. Viabilities of both cell lines were decreased in a dose-dependent manner, with IC_50_ at 33.8 ± 2.6 nM for SW1990 cells and 40.8 ± 3.1 nM for SW1990GR (Figure 5K). Cell proliferation with 0, 25, or 50 nM CYM was monitored by the MTT assay. The results showed that 50 nM CYM significantly decreased the proliferation of SW1990 cells (Figure 5L, left), while 25 nM CYM was sufficient to block the proliferation of SW1990GR cells (Figure 5L, right). Then, SW1990 cells were treated with 30 nM GEM, 25 nM CYM, or 30 nM GEM plus 25 nM CYM for 3 days. Cells exposed to the combined treatment showed a significantly lower viability than cells treated with single agents (Figure 5M). Likewise, CYM significantly decreased the survival of GR cells of another PDAC cell line HPAF-II (Supplementary Figure S5I, left) and a colorectal cancer cell line HT-29 (Supplementary Figure S5I, right). CYM significantly inhibited the expression of *UGT1A10* in both TRA-1-81^+^ and TRA-1-81^–^ subpopulations (Supplementary Figure S5J). Importantly, the ectopic expression of *UGT1A10* partially rescued the anti-chemoresistant activity of CYM (Supplementary Figure S5K). Together, CYM represses *UGT1A10* expression levels (Figure 5I and J), reduces the expression of TRA-1-60/TRA-1-81 (Figure 5H), and, at least partially, eliminates chemoresistant cells (Figure 5K).

### CYM suppresses TRA-1-60 expression and blocks tumor growth *in vivo*

**Figure 6:**
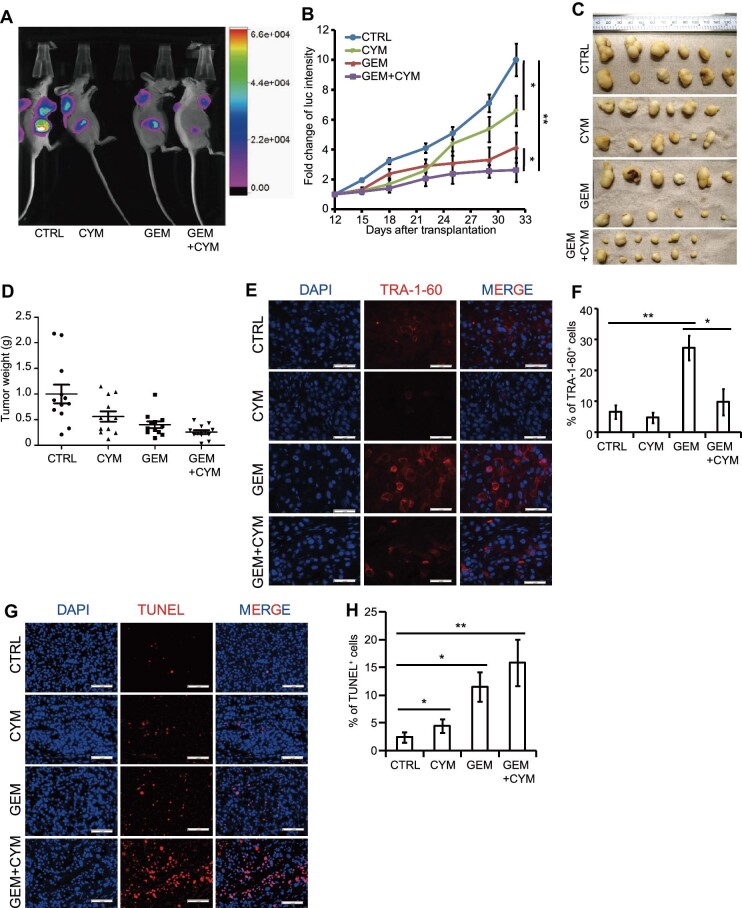
CYM blocks tumor growth and increases chemosensitivity *in vivo*. Mice transplanted with luciferase-labelled SW1990 cells were treated with control, GEM (50 mg/kg bodyweight), CYM (2 mg/kg bodyweight). or GEM (50 mg/kg bodyweight) + CYM (2 mg/kg bodyweight). (**A**) Representative image of mice. (**B**) Growth curve based on luciferase (luc) intensity. (**C** and **D**) Tumor photos (**C**) and weight (**D**). (**E** and **F**) Immunofluorescent staining (**E**) and quantification (**F**) of TRA-1-60^+^ cells in SW1990 xenograft tumors. Scale bar, 50 μm. (**G** and **H**) Immunofluorescent staining (**G**) and quantification (**H**) of TUNEL^+^ cells in SW1990 xenograft tumors. Scale bar, 100 μm. Values in graphs are represented as mean ± SEM. N = 12 independent biological replicates. *P*-values by unpaired two-tailed student's *t*-test were *P < 0.05 and **P < 0.01.

We next sought to validate the effects of CYM *in vivo*, using a xenograft model with luciferase-labelled SW1990 cells propagated in nude mice. Tumor-harboring mice were separated into four groups and treated with PBS (control), 2 mg/kg CYM, 50 mg/kg GEM, or 2 mg/kg CYM plus 50 mg/kg GEM through intraperitoneal injections twice per week. Luciferase intensity was quantified twice per week and normalized with the value obtained from the starting day. After 3 weeks, tumors grew more slowly in the mice treated with GEM or CYM than in control mice, while tumor growth was significantly retarded in the mice treated with GEM plus CYM compared to all other mice (Figure 6A and B; Supplementary Figure S6). Consistent with the imaging, the group of mice treated with GEM plus CYM generated xenograft tumors with the smallest size (Figure 6C) and weight (Figure 6D).

We also tested the effects of CYM on TRA-1-60^+^ cells *in vivo* through examining the percentage of TRA-1-60^+^ cells in xenograft tumors with immunofluorescence. Consistent with the *in vitro* data, CYM administration decreased the percentage of TRA-1-60^+^ cells in GEM-treated tumors from ∼27% to ∼13% (Figure 6E and F). The rate of cell apoptosis in xenograft tumors was assessed with TUNEL assays (Figure 6G and H), which showed that cell death increased in CYM-treated tumors. Additionally, the percentage of TUNEL^+^ cells in tumors exposed to combined treatment was significantly higher than that in tumors treated with GEM or CYM alone. In summary, CYM eliminates TRA-1-60^+^ cells and increases chemosensitivity *in vivo*.

### TRA-1-60/TRA-1-81 expression is positively correlated with short survival and chemoresistance of cancer patients

**Figure 7:**
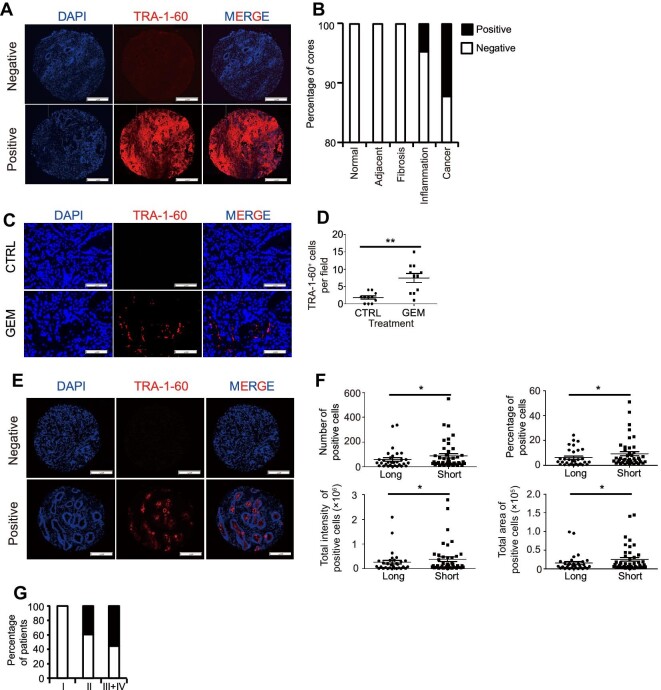
TRA-1-60 specifically stains primary human pancreatic cancer tissues. (**A**) Representative cores negative (upper) and positive (lower) of TRA-1-60 in the commercial TMA. Scale bar, 200 μm. (**B**) Quantitative correlation between TRA-1-60 status of cores and distinct pathologic conditions of pancreatic tissues. (**C** and **D**) Immunofluorescent staining (**C**) and quantification (**D**) of TRA-1-60^+^ cells in patient-derived xenograft tumors in nude mice treated with control or GEM (50 mg/kg bodyweight). Scale bar, 100 μm. (**E**) Representative cores negative (upper) and positive (lower) of TRA-1-60 in the lab-fabricated TMA. Scale bar, 200 μm. (**F**) The number of TRA-1-60^+^ cells, the percentage of TRA-1-60^+^ cells, total TRA-1-60 staining intensity, and total TRA-1-60^+^ area in short and long survival samples. (**G**) Quantitative correlation between TRA-1-60 status and disease progression stage of patients. Values in graphs are represented as mean ± SEM. N = 3 independent biological replicates. *P*-values by unpaired two-tailed student's *t*-test were *P < 0.05 and **P < 0.01.

A primary tissue microarray (TMA) containing pancreatic tissues with defined health conditions was stained with TRA-1-60 antibody. Cores were classified into positive or negative groups (Figure 7A). TRA-1-60^+^ cells were detected in 12% of cancerous cores and <5% of inflammation cores, but undetectable in other types (normal, fibrosis, and cancer-adjacent) (Figure 7B), which further validates the specificity of TRA-1-60 expression.

To examine whether TRA-1-60 expression correlates with chemotherapy treatment in primary tumors, a human pancreatic adenocarcinoma specimen was freshly collected after surgery, transplanted to nude mice, and expanded subcutaneously. Mice carrying the primary human xenograft tumor were separated into two groups: one group was treated with 50 mg/kg GEM twice weekly, while the other group received the same volume of PBS as control. After one month, xenograft tumors were stained with TRA-1-60 antibody. TRA-1-60 staining was detected in GEM-treated xenograft tumors but not in control xenograft tumors (Figure 7C and D), confirming that TRA-1-60 expression is positively correlated with chemoresistance.

Finally, an additional TMA containing 297 cores of pancreatic cancer specimens (99 patients, 3 cores for each patient) was stained with TRA-1-60 antibody (Figure 7E). Based on patient survival history, the samples were separated into short survival and long survival groups. The results showed that 32.7% (17 out of 52) long survival samples and 49.9% (22 out of 45) short survival samples stained positively with TRA-1-60 antibody. We further analyzed the positively stained cores. The number of TRA-1-60^+^ cells, the percentage of TRA-1-60^+^ cells, total TRA-1-60 staining intensity, and total TRA-1-60^+^ area of short survival samples were all significantly higher than those of long survival samples (Figure 7F). The correlation between TRA-1-60 status and disease progression stage of patients was analyzed (Figure 7G). TRA-1-60^+^ cores were not detected in patients with stage I tumors, but detectable among patients at stages II, III, and IV. An increasing trend was found in the percentage of TRA-1-60^+^ patients along the stage progression. Together, the data suggest that TRA-1-60 is a promising marker to predict chemoresistance, progression, and survival of pancreatic cancer patients and potentially helpful to develop antigen-specific targeted therapy.

## Discussion

Identification of specific cell surface markers could benefit targeted chemo- or immuno-therapies. From an antibody-based cell surface marker screen, the common epitope of TRA-1-60/TRA-1-81 was identified as highly enriched in CCCs. TRA-1-60 and TRA-1-81 antibodies are commonly used to label human pluripotent stem cells (Andrews et al., 1984; Shamblott et al., 1998; Takahashi et al., 2007). Expression level of their epitope decreases sharply during stem cell differentiation (Draper et al., 2002). TRA-1-60/TRA-1-81 expression is detected in pancreatic cancer tissues and a small portion of inflamed pancreatic tissue, but not in healthy pancreatic tissue. Considering the specificity of TRA-1-60 and TRA-1-81, minimal side effects are expected for their potential use in targeted therapies.

The common epitope of TRA-1-60 and TRA-1-81 antibodies is an O-glycan structure present in podocalyxin (Schopperle and DeWolf, 2007; Natunen et al., 2011). Sialofucosylated podocalyxin was found to be expressed by metastatic pancreatic cancer cells as a functional ligand of E-selectin and L-selectin (Dallas et al., 2012). In addition, TRA-1-60 was used in a combination of three markers to enrich tumor-initiating cells in human prostate cancer (Rajasekhar et al., 2011). Here, we report a previously unknown role of TRA-1-60 and TRA-1-81 in chemoresistance of multiple cancer types. In addition, TRA-1-60^+^/TRA-1-81^+^ cells show high viability, low apoptosis, and strong capacity of colony and sphere formation in the presence of GEM. The correlation of TRA-1-60 expression with poor survival and chemoresistance of pancreatic cancer patients suggests the value for using TRA-1-60/TRA-1-81 as a marker to predict pancreatic cancer patient survival and response to chemotherapy.

Although TRA-1-60 and TRA-1-81 have been used as pluripotent markers for many years, the mechanism controlling the expression of this antigen is largely unknown. Through global gene profiling, we identified *UGT1A10* as a gene that is highly upregulated in both TRA-1-81^+^ cells and CCCs. Gene knockout and overexpression suggest that UGT1A10 lies within a pathway controlling TRA-1-60 antigen expression. *UGT1A10* is a member of the *UGT1A* cluster, which encodes a family of nine UDP-glucuronosyltransferases (UGTs) to facilitate cellular detoxification and remove aromatic amines. The nine UGTs have unique alternative first exons followed by a set of common exons 2–5 (Owens et al., 1996). Some members of this cluster have been shown to be involved in drug metabolism in the liver and kidney through transferring glucuronic acid to scavenge chemicals (Atsumi et al., 1991; Rivory and Robert, 1995). This gene cluster was reported to regulate chemosensitivity to MTX in breast cancer cell lines (Selga et al., 2009). Furthermore, UGT1A10 was indicated to facilitate detoxification of CPT-11/SN38 in colorectal cancer and lung cancer (Oguri et al., 2004; Cummings et al., 2006; Raynal et al., 2010); however, its role in pancreatic cancer remains unknown. Using sgRNA-based gene knockout and overexpression experiments, we showed that UGT1A10 is both essential and sufficient to control TRA-1-60/TRA-1-81 expression, as well as chemoresistance. Currently, the mechanism how UGT1A10 impacts chemosensitivity, as well as TRA-1-60/TRA-1-81, remains uncertain. Regardless, these results provide a novel insight into the mechanism controlling the expression of TRA-1-60/TRA-1-81 epitope and a platform to identify novel agents that eliminate CCCs.

We discovered CYM, a cardiac glycoside, as a compound able to specifically eliminate TRA-1-60^+^/TRA-1-81^+^ cells. CYM blocks tumor cell growth and decreases chemoresistance both *in vitro* and *in vivo*. Interestingly, a previous screen indicated CYM as a differentiation inducer of human embryonic stem cells (hESCs) (Desbordes et al., 2008), suggesting a conserved mechanism controlling TRA-1-60/TRA-1-81 expression in hESCs and CCCs. Furthermore, we found that CYM decreases *UGT1A10* transcript levels, which might also contribute to its role in reducing chemoresistance. Thus, CYM and possibly other cardiac glycosides might be promising candidate drugs for combinational chemotherapy.

In conclusion, we identified a novel cell surface marker, the epitope of TRA-1-60 and TRA-1-81 antibodies, labelling CCCs with an expression pattern restricted to malignant pancreatic tissues. This marker is an excellent candidate for antigen-specific targeted therapy. Genetic approaches were used to identify UGT1A10 as one potential regulator controlling TRA-1-60/TRA-1-81 expression and chemoresistance in cancer cells, which can be used as a new target for drug discovery. Finally, a high-content chemical screen identified CYM, a cardiac glycoside, which represses *UGT1A10* expression, eliminates TRA-1-60^+^/TRA-1-81^+^ cells, and increases chemosensitivity. UGT1A10 potentially regulates the generation of the ‘stemness’ antigens and presumably other targets that impact chemoresistance. By blocking the expression of *UCT1A10*, CYM enhances chemosensitivity leading to more potent action for GEM. CYM and its analogs may be considered for combinational chemotherapy to achieve better outcomes for pancreatic cancer patients.

For technical reasons, we were unable to determine whether UGT1A10 is a direct target for binding by CYM. Currently, the data show that CYM modulates *UGT1A10* expression levels and this may be through an indirect mechanism. While we showed differential expression *in vivo* of TRA-1-60 for parental and GR tumors using xenografts, we did not analyze directly the growth rates of the tumors during passaging. Suitable antibodies for analyzing UGT1A10 expression in patient tumor samples by immunohistochemistry are currently not available. Finally, while CYM represses *UGT1A10* expression levels, reduces the expression of TRA-1-60/TRA-1-81, and partially eliminates chemoresistant cells, we cannot fully exclude additional mechanisms mediating CYM activity.

## Materials and methods

### Drugs

Gemcitabine hydrochloride (GEM), Cymarin (CYM), irinotecan hydrochloride (CPT-11), and pemetrexed disodium heptahydrate (PMX) were purchased from Sigma-Aldrich.

### Cell lines

Pancreatic cancer cell lines, SW1990, AsPC-1, HPAF-II, and CaPan-2, were purchased from American Type Culture Collection. Lung cancer cell line A549 was from Dr Dingcheng Gao at Weill Cornell Medicine. Prostate cancer cell line DU145 was from Dr Marco Seandel at Weill Cornell Medicine. Colon carcinoma cell line RKO and colorectal carcinoma cell lines HT29 and HCT116 were from Dr Steven Lipkin at Weill Cornell Medicine. All cell lines were maintained in DMEM/F-12 media (Corning) plus 10% fetal bovine serum (FBS; Corning).

To establish GR cell lines, cells were first cultured with 10 nM GEM till cell growth became stable. Then, GEM concentration was progressively increased to 20, 50, and 100 nM. Once cell growth became stable with 100 nM GEM, cells were expanded with 100 nM GEM and used in assays.

### Antibody-based cell surface marker screening

The SW1990 cell line was labelled with EBFP using lentivirus. SW1990EBFP cells were mixed with SW1990GR cells in equal amount and stained with BD Lyoplate Human Cell Surface Marker Screening Panel according to the instruction of the manufacturer. Stained cells were analyzed in BD FACS Calibur cell analyzer controlled by AMS 1.0.2 software (Cytek). Data were quantified with FlowJo 9.8 software.

### Flow cytometry

TRA-1-60 PerCP-efluor710 (eBioscience, 1:50) and TRA-1-81 PE (eBioscience, 1:100) were used. Cells were blocked with 5% FBS plus mouse IgM isotype control antibody (eBioscience, 1:100) at room temperature for 15 min, and then stained on ice for 30 min. Stained cells were resuspended in PBS containing 300 nM DAPI and proceeded to analysis or sorting. Cell apoptosis was analyzed with FITC-Annexin V labelling reagent (BD). Flow cytometry analysis was carried out on BD FACS Calibur with FlowJo 9.8 software. Sorting was performed with FACSVantage SE (Becton Dickinson).

### Immunofluorescent staining

Primary antibodies were TRA-1-60 antibody (BD, 1:200) and biotin-conjugated TRA-1-60 antibody (eBioscience, 1:200). Secondary antibodies were donkey anti-mouse Alexa-efluor594 (Life technologies, 1:500) and streptavidin-conjugated Alexa Fluor® 594 (Life technologies, 1:200).

Cells were fixed with 10% formalin for 20 min at room temperature. After 1 h blocking in PBS with 5% horse serum and 0.3% Triton X-100, primary antibodies were added to cells for incubation overnight at 4°C. Secondary antibody incubation was 1.5 h at room temperature.

Tissue samples were fixed with 4% paraformaldehyde overnight at 4°C, washed with 70% ethanol, and then embedded in paraffin. After de-paraffin and rehydration, antigen retrieval was carried out in hot antigen retrieving buffer with a normal-pressure steamer for 30 min. Blocking was 1 h at room temperature in PBS containing 1% horse serum. Primary antibody staining was overnight at 4°C in PBS plus 0.5% Triton X-100 and 5% horse serum. Secondary antibody incubation was at room temperature for 1.5 h.

### Gene expression profiling

Total RNA samples were extracted from sorted cells with Absolutely RNA Nanoprep Kit (Agilent Technologies) or from cells in culture with miRNeasy Plus Kit (Qiagen). RNA quality was validated with Bioanalyzer (Agilent). cDNA libraries were generated using TruSeq RNA Sample Preparation Kit (Illumina) and sequenced with single reads in HiSeq2500 (Illumina). Gene expression level was analyzed using Cufflinks. Heatmaps were then generated by heatmap.2 in R gplots package. Pathway enrichment analysis was performed online with DAVID functional annotation tool (https://david.ncifcrf.gov/).

### Chemical library screening

The Prestwick library of FDA-approved drugs (∼1280 chemicals) plus a collection of drugs in clinical trials was used for the screen on SW1990GR cells.

For screening, SW1990GR cells were plated in 384-well plates (5000 cells/well). After overnight incubation, chemicals were added at 1 μM or 10 μM in duplicate. After 5 days of treatment, plates were stained with TRA-1-60 antibody and analyzed with MetaXpress High Content Image Acquisition and Analysis System (Molecular Devices). Wells treated with DMSO were set as control. The reduction was calculated by subtracting the percentage of TRA-1-60^+^ cells in the chemical-treated wells from that of control wells. Standard deviation of TRA-1-60^+^ percentage in control wells was calculated and 3-fold of this value was taken as threshold to pick up the primary hit.

### Mouse procedures

To assess *in vivo* effects of CYM, luciferase-labelled SW1990 cells were transplanted to nude mice subcutaneously. Each mouse received four injections (5 × 10^5^ cells/site). Twelve days later, mice were randomly separated into four groups and treated with PBS (control), GEM (50 mg/kg bodyweight), CYM (2 mg/kg bodyweight), or GEM (50 mg/kg bodyweight) plus CYM (2 mg/kg bodyweight) twice weekly via intraperitoneal injection. Image acquisition was performed twice weekly with Xtreme Optical and X-ray small animal imaging system. Luciferin potassium salt (Regis), substrate of luciferase, was applied at 2 mg/25 g bodyweight in 150 μl PBS. Isoflurane (Henry Schein) was used for anesthesia during imaging.

### TMA

The TMA containing normal, non-cancer disease, and cancerous human pancreatic tissues were purchased from Biomax. Another lab-fabricated TMA includes 97 cases (52 long survival and 45 short survival) collected from the archives of Memorial Sloan Kettering Cancer Center, which are pathologically confirmed as PDAC. Three sections from different areas of each tumor were collected. Five normal pancreatic blocks were chosen as controls. The percentages of missing cores for long and short survival groups were indicated by hematoxylin and eosin staining as 14% (22 out of 156) and 2% (3 out of 135), respectively.

### Study approval

All animal experiments were approved by Weill Cornell Medical College Institutional Animal Care and Use Committee in accordance with regulations and guidelines.

All human specimens used were approved by Weill Cornell Medical College Institutional Review Boards. Two appropriate informed consent forms have been signed by PDAC patients.

### Statistical analysis

Statistical significance was determined by Student's *t*-test (one-tailed) or analysis of variance (two-way). The significance was defined as *P < 0.05 or **P < 0.01.

## Supplementary Material

mjad039_Supplemental_File

## Notes

**Author contributions:** S.C. and T.E. designed research; L.T., X.D., P.M., T. Zhou, T.I., and S.A. performed research; L.T., T. Zhang, and Z.X. performed bioinformatics analysis; B.H., G.A., F.M., M.D.L., C.A.I., and S.D.L. provided primary tissues and tissue assay; L.T., T.E., and S.C. analyzed data; and L.T., X.D., G.A., S.D.L., S.C., and T.E. wrote the manuscript.
