# Supplementary material for: A targetable pathway to eliminate TRA-1-60+/TRA-1-81+ chemoresistant cancer cells
Source: J Mol Cell Biol. 2023 Jun 16;15(6):mjad039. doi: 10.1093/jmcb/mjad039 (PMC10847630; doi:10.1093/jmcb/mjad039)
Supplement: mjad039_Supplemental_File [file mjad039_supplemental_file.pdf]

Supplementary Experimental procedures

Constructs

pLV-EBFP2-nuc plasmid (Addgene #36085) was used to label SW1990 cells for cell surface marker screening.

LentiLuc-blast and LentiUGT1A10-blast plasmids were generated based on LentiCas9-blast vector (Addgene, 52926). *Luciferase*c DNA was cloned from pGL3-promoter plasmid (Promega) with PCR amplification and inserted between *Age*I and *Bam*HI sites. *UGT1A10* cDNA was PCR amplified from plasmid purchased from Dharmacon (MHS6278- 202756114) and inserted between *Xba*I and *Bam*HI sites. Primers were used as below:

Luc-Forward: accggtATGGAAGACGCCAAAAACATAAAG;

Luc-Reverse: ggatccCACGGCGATCTTTCCGCCCTTCTT;

UGT1A10-Forward: tctagaATGGCTCGCGCAGGGTGGA;

UGT1A10-Reverse: agatctATGGGTCTTGGATTTGTGGGCTTT.

Plasmids for knockout were cloned based on LentiCRISPRv2 vector (Addgene #52961) between two *Bsm*BI sites (NEB, R0580S) with online guidance (https://www.addgene.org/static/data/plasmids/52/52961/52961-attachment_B3xTwla0bkYD.pdf). The sequence of sgRNAs are listed below:

CCL26-1: CAGTTCCAAGGTGGAGACTC;

CCL26-2: TTCCAAGGTGGAGACTCAGG;

KRT6b-1: GGAACGGGGTCGTCTGGACT;

KRT6b-2: AGGCAGCTGGACAACATCGT;

NFASC-1:GTCAGCGAAGGATCACATCG;

NFASC-2: TCAATCAGGATGTTATCACG;

SLC2A9-1:CTACGGCTACAACCTGTCGG;

SLC2A9-2:TGGGGGCATTCACCACCGAC;

UGT1a10-1:TGGTTTTCGCCCATGCTCAA;

UGT1a10-2:CTATTAATGAGTTCATCCAG;

UGT1a6-1:GAGGTATCAACTGTAAGAAG;

UGT1a6-2: ACCTCCAATGAAGACCATGT;

ZNF704-1:GAGGACTTAAAACGTGACTG;

ZNF704-2: TGCTGGAGGAACATCAATGT;

CTRL1: GGGCGAGGAGCTGTTCACCG;

CTRL2: GAGCTGGACGGCGACGTAAA.

Cell viability, colony formation, sphere formation and growth curve

To quantify viability, cells were plated into 96-well plates (6000/well) one night before treatment. Drugs were then added in indicated dose for 5 days. After fixation, cell nuclei were stained with DAPI (Invitrogen). Plates were analyzed using MetaXpress High Content Image Acquisition and Analysis System (Molecular Devices).

For colony formation, cells were seeded to 6-well plates (2000/well) with or without GEM. Medium was refreshed every 2 days. Four days later, GEM-free media was applied to all wells for another 4 days of culture. The plates were then stained with 20 mg/ml crystal violet (SigmaAldrich) for 30 min. After washing with running water, the colony numbers were counted.

For sphere formation, cells were plated to ultra-low attach 6-well plate (2000/well) in DMEM/F-12 supplemented with 1X B27, 20 ng/ml bFGF and 20 ng/ml EGF plus given dose of GEM. 8 days later, number of spheres was counted under a microscope.

To quantify growth, Cells were seeded into 96-well plates (2000/well). After overnight incubation, medium with indicated drug was refreshed. Cell proliferation was quantified every 2 days using CellTiter 96® AQueous Non- Radioactive Cell Proliferation Assay (Promega).

qRT-PCR

Reverse transcription reaction was prepared with 0.5-2 μg total RNA using High Capacity cDNA Reverse Transcription Kit (Applied Biosystems). Products were diluted 10 folds with DNase&RNase free water as template. qPCR reactions were prepared with 2X LightCycler 480 SYBR Green I Master reagent (Roche) and proceeded in LightCycler 480 II (Roche) with software LightCycler 480 SW1.5.1 for signal acquisition and processing. *ACTB* was employed as internal reference. Primers were listed below:

UGT1A10-Forward: GACTATCCCAAACCCGTGATG;

UGT1A10-Reverse: CCCAAAGAGAAAACCACAATTCC;

ACTB-Forward: ACCTTCTACAATGAGCTGCG;

ACTB-Reverse: CCTGGATAGCAACGTACATGG.

Western blot

Cells were lyzed in complete RIPA buffer (Millipore). Lysates were loaded on 4-12% Bis-Tris gels (Life technologies). After electrophoresis, protein was transferred to nitrocellulose membrane (Life technologies) and blocked with 5% milk in TBST, then incubated with primary antibody overnight.

The antibodies are mouse anti- UGT1A10 polyclonal antibody (Abcam, ab194474) and mouse anti-GAPDH antibody (Abcam, ab8245). The membrane was washed and incubated with secondary antibody, peroxidase Horse Anti-Mouse IgG Antibody (Vector Labs), in TBST containing 5% milk for 1 hour at room temperature. Membrane was developed with Chemiluminescent Substrate (LI-COR) and scanned with C-DiGit Blot Scanner (Licor) and analyzed with Image Studio Digits 4.0 software.

Supplementary Table S1. Candidate genes for functional validation. Related to Figure 3.

ID	Name	Experiment 1			Experiment 2			Acquired chemoresistance		
		Expression level		Fold	Expression level		Fold	Expression level		Fold
		TRA-1-81^{-}	TRA-1-81^{+}		TRA-1-81^{-}	TRA-1-81^{+}		SW1990	SW1990GR	
NM_020041	SLC2A9	1.21	3.37	2.78	1.62	4.34	2.68	0.19	11.22	58.09
NM_001033723	ZNF704	0.27	0.77	2.80	0.22	0.60	2.75	0.03	3.04	114.15
NM_005555	KRT6B	0.43	2.29	5.27	0.31	1.12	3.58	0.06	5.67	102.11
NM_019075	UGT1A10	0.45	1.54	3.44	0.31	1.21	3.89	0.59	98.58	167.22
NM_006072	CCL26	0.12	0.47	3.96	0.07	0.27	4.15	0.19	35.60	188.56
NM_015090	NFASC	0.08	0.33	4.27	0.04	0.18	4.15	0.01	4.44	337.58
NM_001072	UGT1A6	0.66	6.69	10.09	0.62	6.36	10.29	0.00	57.10	1.99E+05

Supplementary Figure S1

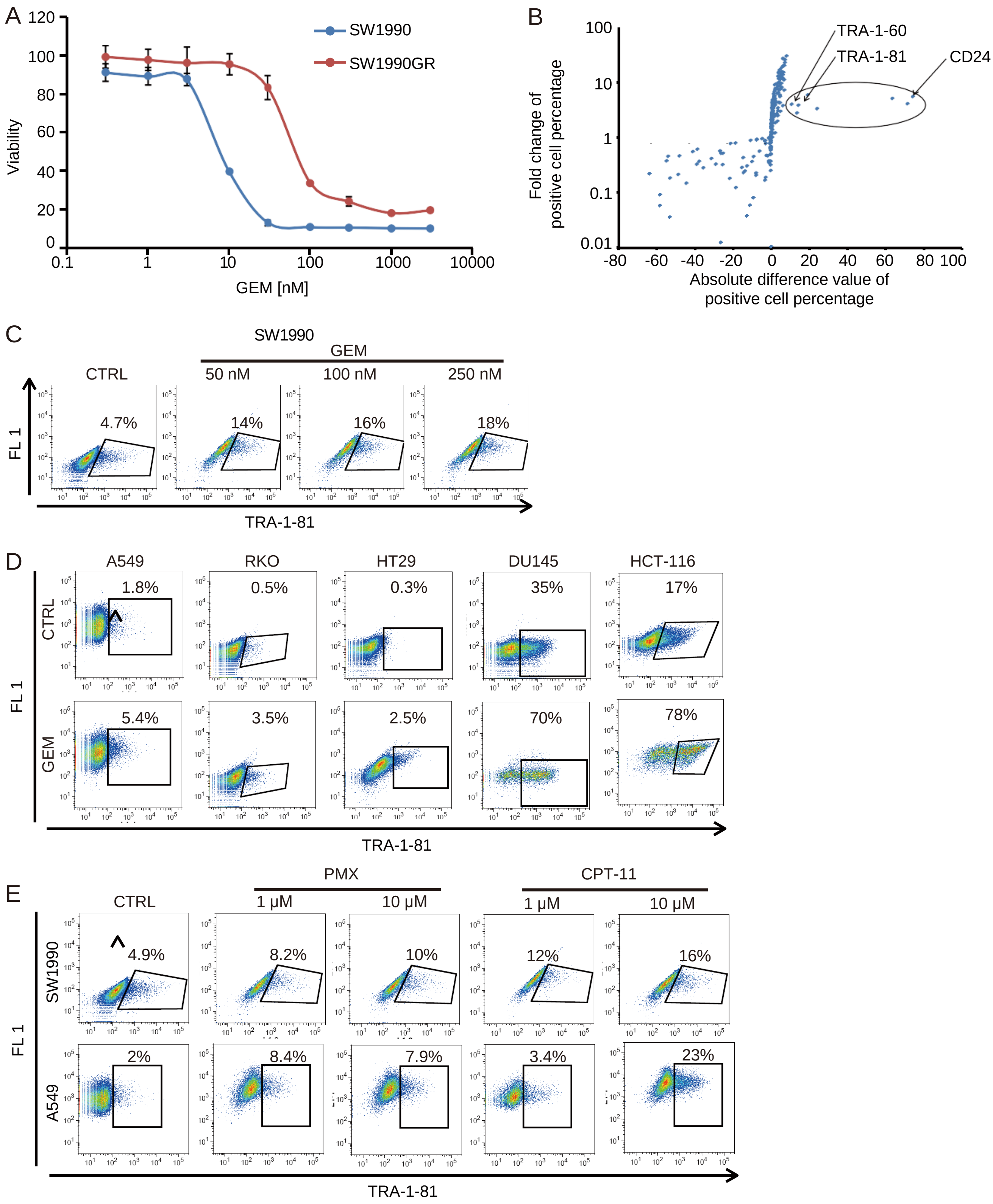

Figure S1. TRA-1-60 and TRA-1-81 are enriched in chemoresistant cancer cells. Related to Figure 1.
(**A**) Viability curve of SW1990 and SW1990GR cells in presence of GEM. (**B**) Results of primary cell surface marker antibody library screening. X axis presents the absolute difference value, which is calculated by subtracting the percentage of positive cells in SW1990 cells from that of SW1990GR cells. Y axis presents the fold change, which is calculated by dividing the percentage of positive cells in SW1990GR cells by that of SW1990 cells. (**C**–**E**) Flow cytometry plots of TRA-1-81 expression acute drug treatment. (**C**) Flow cytometry plots of TRA-1-81 expression in SW1990 cells treated with GEM, which are quantified in Fig. 1G, (**D**) Flow cytometry plots of TRA-1-81 in multiple cancer cell lines treated with GEM, which are quantified in Fig. 1H, (**E**) Flow cytometry plots of TRA-1-81 in SW1990 (upper) and A549 (lower) cell lines treated with PMX or CPT-11, which are quantified in Fig.1I, J. Values in graphs are represented as Mean of 3 independent biological replicates.

Supplementary Figure S2

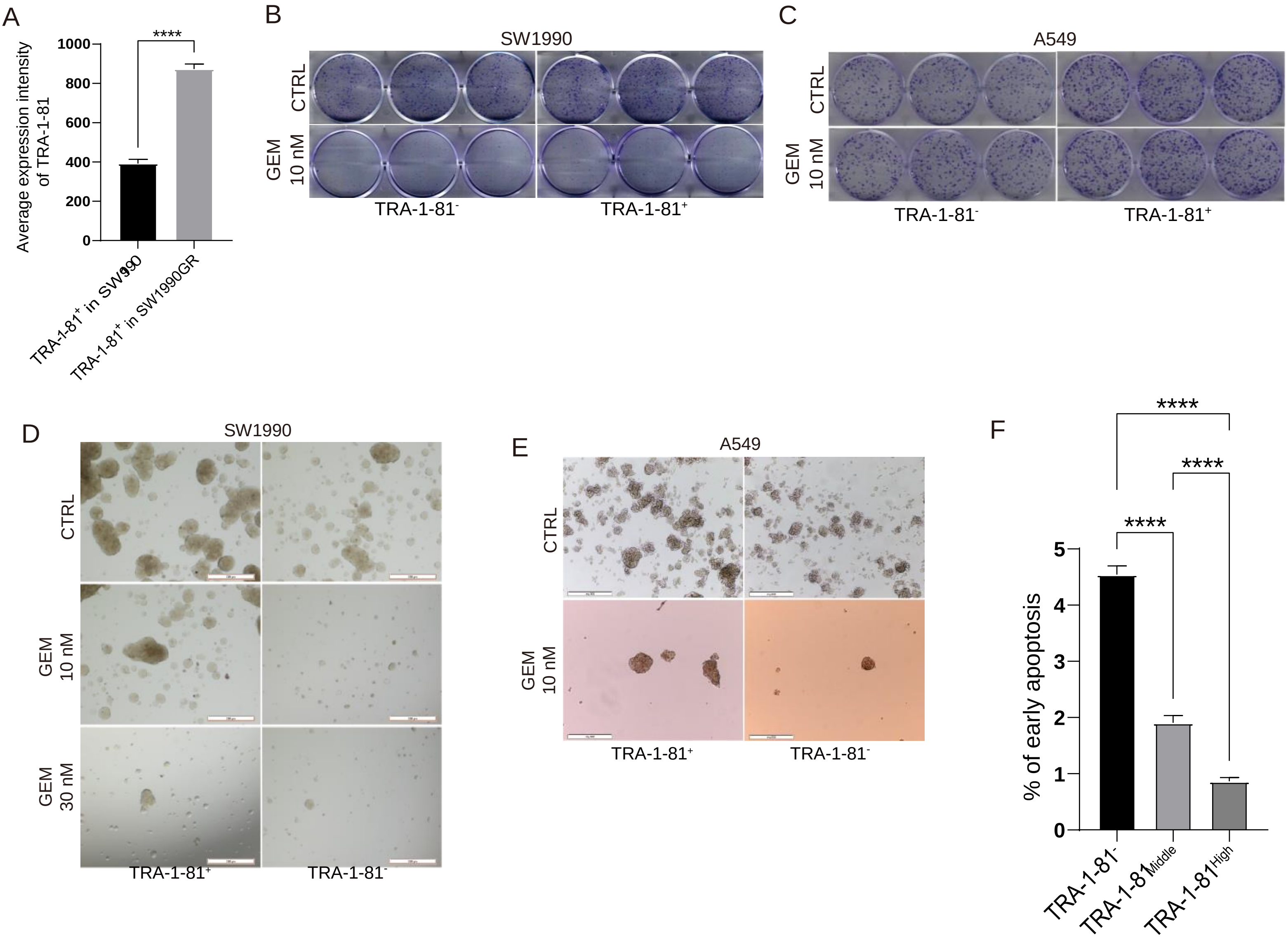

Figure S2. Images of colony formation and sphere formation. Related to Figure 2.

(A) Quantification of average expression intensity of TRA-1-81 in TRA-1-81$^+$ SW1990 and TRA-1-81$^+$ SW1990GR cells. (B) Phase contrast images of colonies, which is quantified in Fig. 2C. (**C**) Phase contrast images of colonies, which is quantified in Fig. 2D. (**D**) Phase contrast images of spheres, which is quantified in Fig. 2E. (**E**) Phase contrast images of spheres, which is quantified in Fig. 2G. Scale bar, 500 μm. (**F**) Quantification of early apoptosis rate in TRA-1-81$^-$, TRA-1-81^{Middle} and TRA-1-81^{High} in SW1990 cells treated with GEM.

Supplementary Figure S3

A

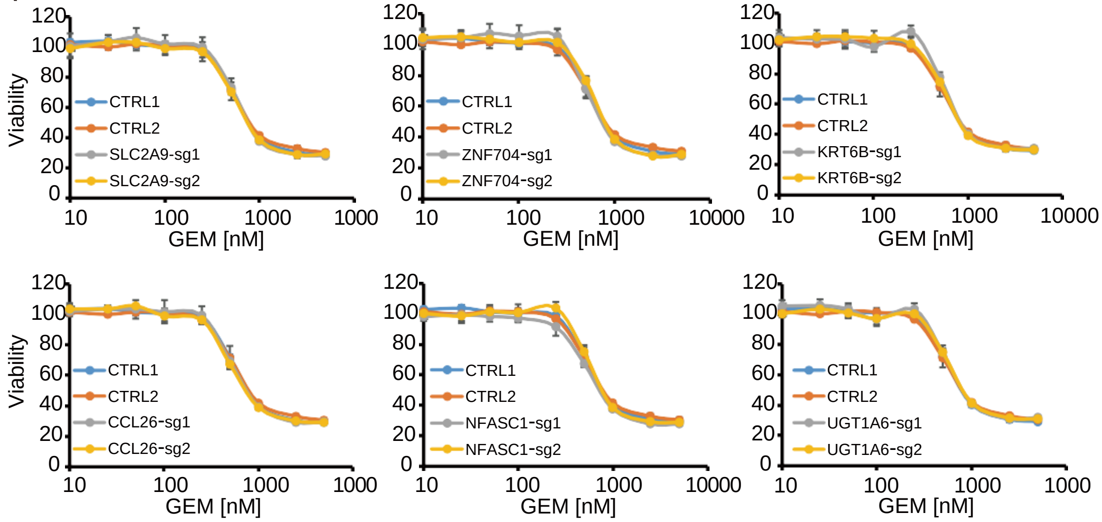

B

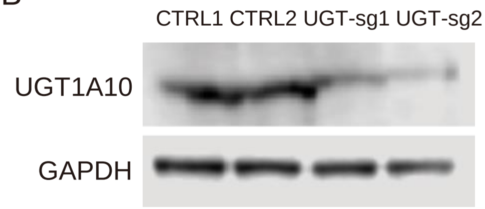

C

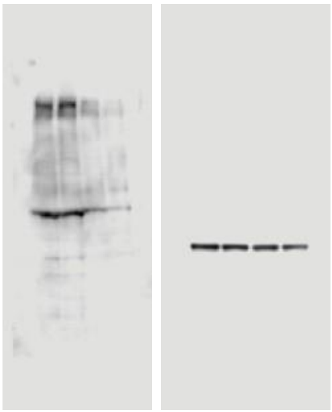

Fig S3. The effect of knockout of candidate genes. Related to Figure 3. (**A**) Viability curve of SW1990GR cells carrying sgRNAs of six candidate genes: SLC2A9, ZNF704, KRT6B, CCL26, NFASC1 and UGT1A6. (**B**) Western blot analysis of UGT1A10 expression in UGT-sg1 and UGT-sg2 cells. (**C**) Original western blot images of UGT1A10 (left) and GAPDH (right). Values in graphs are represented as Mean±SEM. N=3 independent biological replicates.

Supplementary Figure S4

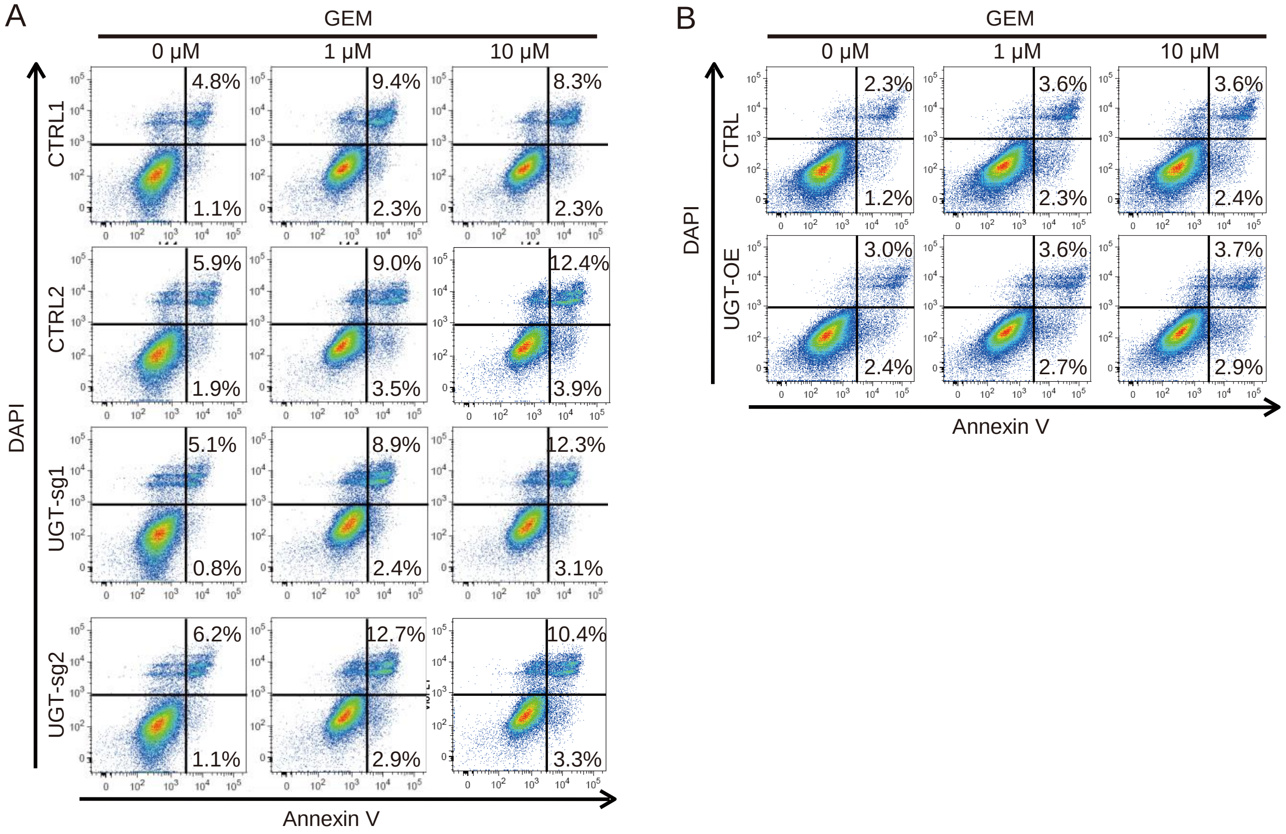

Fig S4. Flow cytometry analysis of apoptosis in GEM treated UGT-sg cells or UGT-OE cells. Related to Figure 4. (**A**) Flow cytometry analysis of cell apoptosis of UGT-sg cells, which is quantified in Figure 4B. (**B**) Flow cytometry analysis of cell apoptosis of UGT-OE cells, which is quantified in Figure 4F. Values in graphs are represented as Mean of 3 independent biological replicates.

Supplementary Figure S5

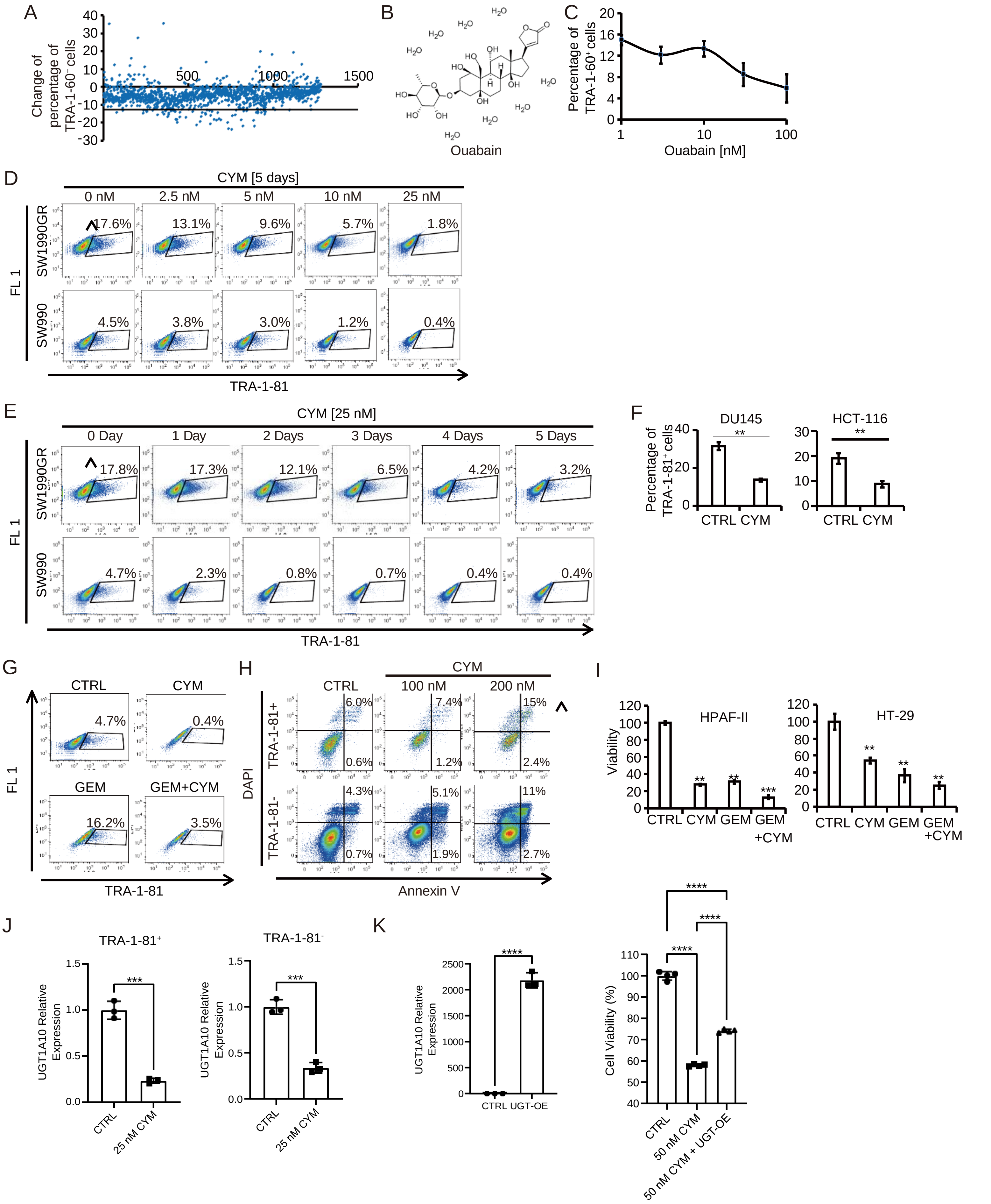

Figure S5. Primary chemical screening results and effects of CYM. Related to Figure 5.
(**A**) Scatter plot of primary chemical screening results. (**B**) Chemical structure of Ouabain. (**C**) Inhibitory curve of Ouabain. (**D**) Flow cytometry plots of TRA-1-81 expression of cells treated with different doses of CYM. Upper panel was quantified in Fig. 5D and lower panel in Fig. 5E. (**E**) Flow cytometry plots of TRA-1-81 expression in cells with different days of 25 nM CYM treatment. Upper panel was quantified in Fig. 5F and lower panel in Fig. 5G. (**F**) The percentage of TRA-1-81+ population in other cancer cell lines, such as DU145 (left) and HCT116 (right), under 25 nM CYM treatment for 5 days. (**G**) Flow cytometry plots of TRA-1-81 expression of cells treated with 100 nM GEM, 25 nM CYM or 100 nM GEM plus 25 nM CYM. Quantified in Fig. 5H. (**H**) Flow cytometry plots of Annexin V staining in SW1990GR cells after CYM treatment. (**I**) Viability of cancer cell lines HPAF-II (left) and HT-29 (right) treated with 50 nM GEM and 25 nM CYM for 5 days. (**J**) qRT-PCR analysis of UGT1A10 expression in TRA-1-81- and TRA-1-81+ SW1990GR cells after 25 nM CYM treatment. (**K**) Cell viability of control and UGT1A10 overexpressing SW1990 cells treated with 50 nM CYM. Values in graphs are represented as Mean±SEM of 3 independent biological replicates. p values by unpaired one-tailed student t-test were $^{**}p<0.01$; $^{***}p<0.001$.

Supplementary Figure S6

A

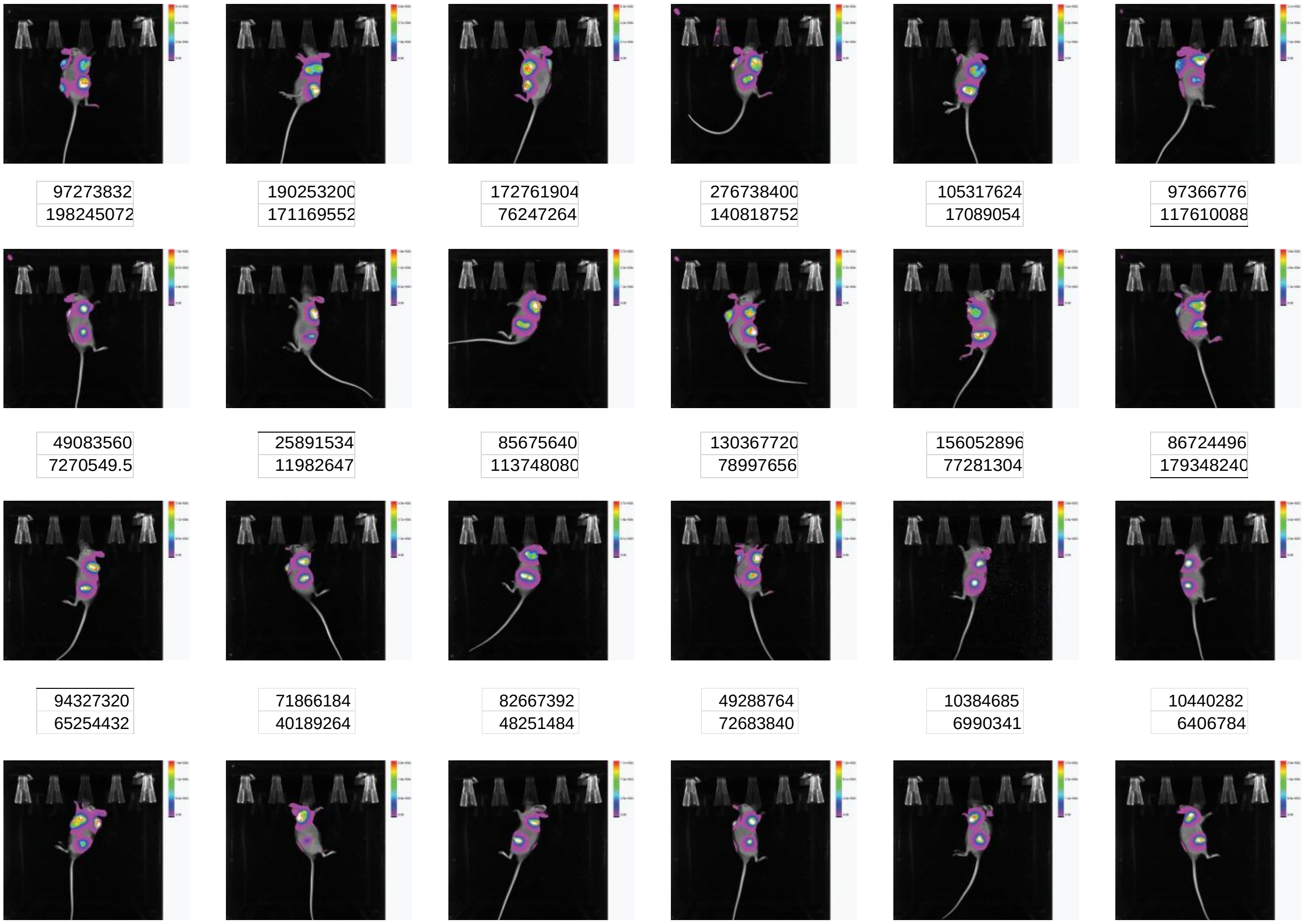

CTRL Sum Intensity	97273832	190253200	172761904	276738400	105317624	97366776
	198245072	171169552	76247264	140818752	17089054	117610088
CYM Sum Intensity	49083560	25891534	85675640	130367720	156052896	86724496
	7270549.5	11982647	113748080	78997656	77281304	179348240
GEM Sum Intensity	94327320	71866184	82667392	49288764	10384685	10440282
	65254432	40189264	48251484	72683840	6990341	6406784
GEM+CYM Sum Intensity	90047752	72817560	26758996	17968896	56197032	87074456
	2520267	12778066	8940976	17963360	53509412	61159400

Figure S6. Representative image and summary intensity of mice transplanted with luciferase labeled-SW1990 cells treated with control, GEM (50 mg/kg bodyweight), CYM (2 mg/kg bodyweight) or GEM (50 mg/kg bodyweight) plus CYM (2 mg/kg bodyweight). Related to Figure 6.
